# Late Effects of Clubfoot Deformity in Adolescent and Young Adult Patients Whose Initial Treatment Was an Extensive Soft-tissue Release: Topic Review and Clinical Case Series

**DOI:** 10.5435/JAAOSGlobal-D-19-00126

**Published:** 2020-05-01

**Authors:** Jeffrey E. Johnson, Thomas A. Fortney, Pamela C. Luk, Sandra E. Klein, Jeremy J. McCormick, Matthew B. Dobbs, J. Eric Gordon, Perry L. Schoenecker

**Affiliations:** From the Department of Orthopaedic Surgery, Washington University School of Medicine, St. Louis, MO (Dr. Johnson, Dr. Klein, Dr. McCormick, Dr. Dobbs, Dr. Gordon, Dr. Schoenecker); the Department of Orthopaedic Surgery, Dartmouth-Hitchcock School of Medicine, New Hampshire, NH (Dr. Fortney); and the Congress Orthopaedic Associates, Arcadia, CA (Dr. Luk).

## Abstract

Children with congenital clubfoot often have residual deformity, pain, and limited function in adolescence and young adulthood. These patients represent a heterogeneous group that often requires an individualized management strategy. This article reviews the available literature on this topic while proposing a descriptive classification system based on a review of patients at our institution who underwent surgery for problems related to previous clubfoot deformity during the period between January 1999 and January 2012. Seventy-two patients (93 feet) underwent surgical treatment for the late effects of clubfoot deformity at an average age of 13 years (range 9 to 19 years). All patients had been treated at a young age with serial casting, and most had at least one previous surgery on the affected foot or feet. Five common patterns of pathology identified were as follows: undercorrection, overcorrection, dorsal bunion, anterior ankle impingement, and lateral hindfoot impingement. Management pathways for each group of the presenting problems is described. To our knowledge, this topic review represents the largest report of adolescent and young adult patients with residual clubfoot deformity in the literature.

The treatment for infants and young children with congenital clubfoot deformity has been, and continues to be, studied extensively.^1,2,3,4,5,6,7,8,9,10,11,12,13,14^ Treatments continue to evolve, with recent studies indicating that serial casting techniques with judicious use of surgery provide better long-term results than early extensive soft-tissue release.^2,6,7,9,10^ Regardless of the treatment method, patients with congenital clubfoot can have abnormalities in the foot structure and function that affect them into adulthood.^1,10,12,13,15^

Reported late sequelae of treated congenital clubfoot deformities include recurrent or residual deformity (cavus, heel varus,and forefoot adduction),^1,2,3,4,5,6,7,8,9,10,11,12,13,14,15,16,17,18,19,20,21^ pes planovalgus deformities,^1,4,5,18-20^ pain,^1,3,6,7,10,12,15,17,18^ limited ankle and subtalar range of motion,^1-3,7,8,10,12,14-16,18,20^ limitation of activities,^1,2,7,12,15,18^ abnormal gait,^3,7,15,18^ small foot,^7,12,20^ dorsal bunion,^4,18,22^ abnormal ankle architecture,^1,7,16^ navicular abnormalities,^1,7,16,18,21^ weakness,^1,4,7^ altered plantar pressures, degenerative joint changes, limitation of shoe wear,^7,15,17-19,21^ cock-up first toe,^14^ pseudoaneurysm,^18^ and talar collapse.^18^ Although patients with congenital clubfoot often do not have normal feet after treatment, literature pertaining to adolescent or young adult patients with a history of congenital clubfoot is scarce.

Few reports exist in the literature concerning patients in their second or third decades of life with clubfoot sequelae, despite the fact that these patients are seen fairly often within our institutions.^17,20,23,24^ This article reviews the available literature on the surgical treatment of adolescent and young adult patients with persistent clubfoot pathology while proposing a descriptive classification system based on the review of patients who have undergone surgery for this problem at our institutions. Five common patterns of pathology were identified in this patient population. Identification of these patterns is helpful in the evaluation of the late sequelae of previous treatment for clubfoot deformities and for the development of management pathways for each group of presenting problems.

These problems resulting from extensive soft-tissue release are in contrast to the Ponseti Method that has proven excellent long-term results into adulthood.^9,10,15^

## Methods

A retrospective chart review was performed to identify the various types of problems that occur in the adolescent and young adult population after clubfoot treatment as a child. Databases at two institutions (a pediatric hospital and a large academic medical center) encompassing the practice of five orthopaedic surgeons were searched from the dates January 1999 to January 2012. Inclusion criteria were as follows: patients were treated during infancy and/or childhood for clubfoot deformity—treatments could include any combination of operative and nonsurgical interventions. All types of congenital clubfoot were included. Patients who were between 9 and 19 years of age at the time they underwent surgery for problems related to a previous clubfoot deformity during the period between January 1999 and January 2012 were retrospectively identified. Patients with neglected clubfoot deformities or with developmental deformities (ie, patients with a previously normal foot) were excluded.

Complete patient histories, including previous treatments, presenting complaints, and the details of the surgical treatment were recorded. Clinical outcomes data were not available in enough patients to make meaningful conclusions regarding the success of treatment.

The types of presenting diagnoses were examined for similarities in an attempt to identify any common patterns within our patient population. Patients were subsequently grouped into categories of deformity and surgical intervention. Mean age and number of previous procedures were calculated for all patients and for each category. No formal statistical analyses were performed secondary to the varied nature of the patient population, quantity of available data points for each patient, and small numbers within several groups.

## Results

During the study period, 72 patients (93 clubfeet) underwent surgery related to previous clubfoot deformity at an average age of 12.9 years (range 9 to 19 years). None of these patients were treated with the Ponseti method for the treatment of clubfoot with casting, Achilles tenotomy, and bracing treatment. All except 3 patients had undergone previous clubfoot surgery, typically before 1 year of age. On average, patients had undergone 2.24 previous operative interventions per foot and over 60% had multiple surgeries before the target surgery. All patients in the series presented with foot deformity and pain in adolescence or young adulthood. A total of 201 procedures (2.16 per foot) were performed at the time of their index surgical reconstruction to correct the sequalae of previous treatment.

**Figure 1:**
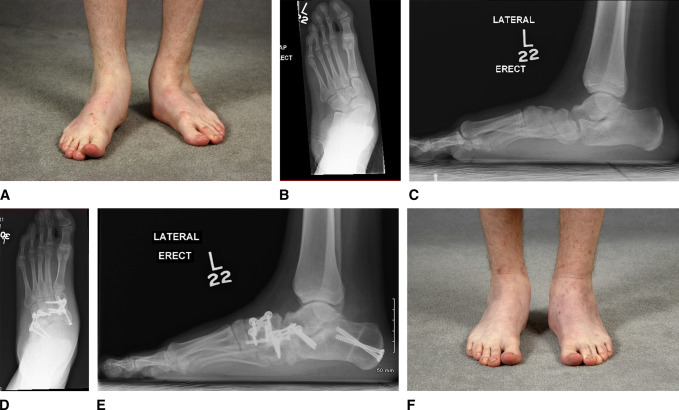
Overcorrection. **A**–**F**, Preoperative and postoperative radiographs and photographs of a 14-year-old boy with a history of bilateral congenital clubfoot, who underwent extensive soft-tissue releases at 6 months of age. He presented with pes planovalgus deformity consistent with overcorrection. He was treated with a medial displacement calcaneal osteotomy, calcaneocuboid joint bone-block distraction fusion, midtarsal fusion, advancement of the posterior tibialis tendon, spring ligament reefing, open tendoachilles Z-lengthening, and intramuscular lengthening of peroneus longus and peroneus brevis.

**Figure 2:**
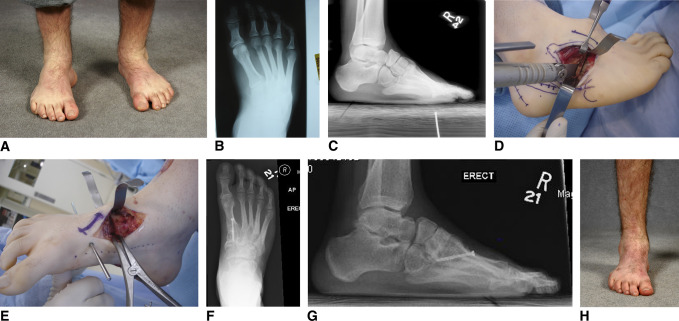
Undercorrection. **A**–**H**, Preoperative and postoperative radiographs and photographs of a 17-year-old boy with a history of idiopathic clubfoot and an extensive soft-tissue release at 6 months of age, presenting with recurrent (undercorrected) deformity. The deformity was centered around the midfoot. He was treated with an open plantar fascia release, biplanar midfoot osteotomy with lateral closing and medial opening wedge, dorsiflexion osteotomy of the first metatarsal, anterior tibialis tendon transfer to the third cuneiform, and a peroneus longus to peroneus brevis tendon transfer.

**Figure 3:**
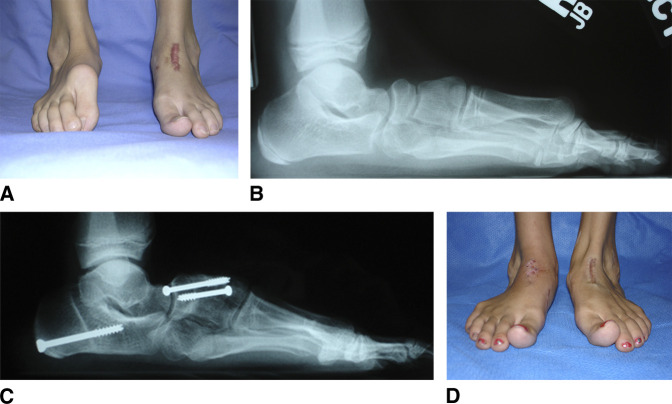
Dorsal bunion. **A**–**D**, Preoperative and postoperative radiographs and clinical photographs of a 10-year-old girl with a history of bilateral congenital clubfoot treated with extensive soft-tissue releases at 6 months of age, who presented with bilateral dorsal bunion deformities. She was treated with a double bone-block fusion of the navicular-first cuneiform and first-second intercuneiform joints, anterior tibial tendon transfer to the second cuneiform, flexor hallucis brevis intramuscular lengthening, first metatarsophalangeal joint release and pinning, and flexor hallucis longus tendon transfer to the first metatarsal neck. The photographs illustrate the preoperative deformity on the right foot after left foot correction and postoperative correction of both feet.

**Figure 4:**
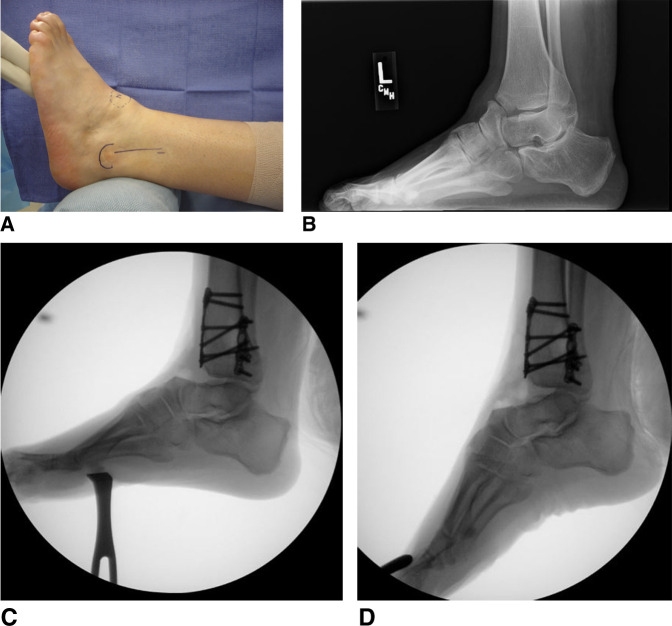
Anterior Impingement. **A**–**D**, Preoperative, intraoperative, and postoperative radiographs and preoperative clinical photographs of an 11-year-old boy with a history of congenital clubfoot and multiple previous surgeries. His primary report was anterior ankle pain and inability to achieve neutral dorsiflexion. Residual cavovarus deformity was also present. He was treated with a dorsiflexion-producing anterior closing-wedge tibial osteotomy with fibular osteotomy, midfoot osteotomy, plantar fascia release, and anterior tibialis tendon transfer to second cuneiform.

**Figure 5:**
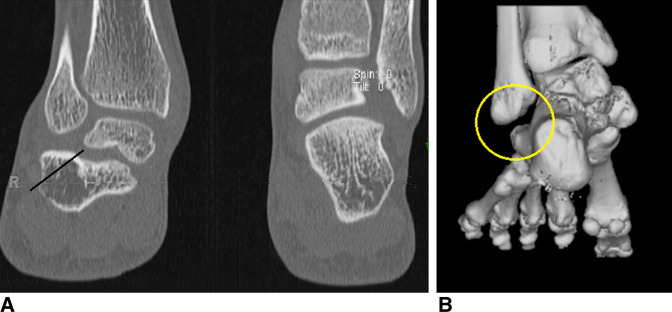
Lateral Impingement. **A** and **B**, Preoperative CT scan of a patient with a history of congenital clubfoot deformity and report of lateral hindfoot pain. The CT scan demonstrates subfibular impingement of the lateral calcaneus. Treatment included a lateral calcaneal wall exostectomy and débridement of the subfibular region. The black line represents an approximation of the bone removal.

With evaluation of the types of complaints and surgeries pertaining to this patient population, the authors noted five common patterns of pathology: overcorrection, undercorrection, dorsal bunion, anterior ankle impingement, and lateral impingement. Patients with pes planovalgus or similar deformities were termed overcorrection and typically underwent flatfoot-type reconstructive procedures (Figure 1). Patients with cavovarus or similar residual deformities were termed undercorrection and typically underwent cavus-foot-type reconstructive procedures (Figure 2). Patients with an elevated first ray and forefoot varus were assigned the category of dorsal bunion. Many of these patients underwent a double bone-block midfoot fusion,^24^ with or without tibialis anterior tendon transfer (Figure 3). Those termed anterior impingement (Figure 4) had abutment of either the talar neck or navicular against the tibia and subsequent limitation in dorsiflexion. These patients underwent débridement of the navicular, a dorsiflexion-producing anterior closing-wedge tibial osteotomy or a combination. Lateral impingement/other category (Figure 5), included three patients with lateral subfibular bony impingement. These patients underwent a lateral wall calcaneal exostectomy. The fourth patient in the lateral impingement/other category had a leg-length discrepancy and underwent distal femoral epiphysiodesis.

The number of procedures performed during the target surgery were calculated and reported for each foot involved in the study. As a whole, patients underwent an average of 2.16 procedures at the target surgery. Patients in the dorsal bunion and undercorrection categories tended to undergo more complex target surgeries, with an average of 3.00 and 2.81 procedures performed per foot, respectively. The average number of procedures at the target surgery has also been reported in Table 1 (Supplement, http://links.lww.com/JG9/A74). These findings may be useful in demonstrating the level of complexity involved in correcting deformities in each category.

**Table 1:** 

Diagnosis Category	Average No. of Previous Procedures	Average No. of Procedures at Target Surgery
Anterior impingement	2.44	1.38
Overcorrection	2.27	1.78
Undercorrection	2.30	2.81
Dorsal bunion	1.80	3.00
Lateral impingement/other	2.17	1.25
All categories	2.24	2.16

Average number of procedures performed previously for each category of deformity and the number of procedures required at the index surgery to correct the deformity.

## Literature Review

We limited our literature review to the management of recurrent and residual clubfoot deformity in adolescent patients treated with previous surgery. The references in the literature concerning this patient population are few, and most articles focus on a single type of deformity seen in residual clubfoot or discuss 1 particular treatment method. Existing articles include relatively small sample sizes, and many rely on subjective and nonvalidated outcome measures. Meaningful data that can lead to conclusions on clinical outcomes are difficult to obtain because of the wide variability in the types and severity of deformities and treatments used.

The late effects of clubfoot deformity in adolescents and young adulthood present a multitude of management challenges, as described in this report and others.^7,12,17,23-25^ Residual problems related to a previous clubfoot deformity may take many forms. The treatments of infants and young children with congenital clubfoot continue to be refined and have evolved over time. Current articles, within the past decade, suggest that for initial treatment of a clubfoot deformity, appropriate casting and/or stretching techniques combined with limited surgical soft-tissue releases may lead to better results than casting combined with extensive soft-tissue releases.^2,6,7,9,10,13^ Even the latest techniques can still result in long-term sequelae of the deformity in some patients.^1,10,12,13,15^ In the older patient with a recurrent or persistent deformity, the foot and ankle is often characterized by joint stiffness, nonpassively correctable deformity, developmental periarticular angular deformities, and bony impingements. Therefore, treatments in these older patients rely less on serial casting techniques and more on surgical correction of these late effects.

## Undercorrection

Relapse of a previously corrected clubfoot or residual deformity resulting from incomplete correction can each result in an undercorrected clubfoot. Relapse and residual deformities are common topics in the literature concerning previously operated clubfeet, but often no distinction is made between these entities.^16,19,21,26-28^ Most articles included a small number of patients and a single treatment strategy.^19,21,27,28^ In cases of undercorrected clubfeet, revision surgery often includes soft-tissue releases with or without the addition of fixation hardware, corrective osteotomy, or arthrodesis.^17,20^ Soft-tissue releases are used more often in younger patients and those with mild, flexible deformities, whereas osteotomies and fusions are reserved for older patients with more severe, rigid deformities.^17,20,22,26^

Atar et al^16^ reported acceptable results in 27 of 29 feet undergoing revision clubfoot surgery, with 19 achieving good or excellent outcomes. The patients in this study ranged from 1 to 12 years in age. The most common procedure performed in this series was revision complete soft-tissue release, alone or in conjunction with plantar release, calcaneocuboid fusion, and navicular-medial cuneiform-first metatarsal joint capsulotomies.^16^ The authors suggested a thoughtful treatment algorithm based on patient age and deformity present, but outcomes were based on a subjective rating system.

Chu et al^26^ published the results of revision soft-tissue release and calcaneocuboid arthrodesis performed in 20 patients (27 clubfeet) for recurrent clubfoot deformity. Patients ranged from 5 to 8 years of age at the time of the arthrodesis, and all had undergone previous clubfoot surgery. Although all patients were initially evaluated at an average of 5.5 years postoperative, 10 patients were re-evaluated at a mean follow-up of 17.5 years. During the interval period between evaluations, functional outcome scores decreased in all patients. Still, the authors note that the final results are comparable with that of other studies on patients undergoing revision clubfoot surgery.^26^

Malizos et al^19^ reported on 13 relapsed clubfeet in 12 patients treated at a mean age of 5.7 years (range 1.5 to 17 years) with revision soft-tissue release and Kirschner wire fixation or the addition of the Ilizarov frame. All patients had undergone previous soft-tissue release, but no bony procedures. In total, 10 cases were idiopathic clubfoot, whereas sciatic nerve palsy, myelomeningocele, and encephalomyelitis were predisposing factors in the remaining three patients. The average Laaveg-Ponseti score for all patients was 86.7 (maximum of 100), whereas the 7 patients treated with an Ilizarov frame had a mean score of 85.1. Radiographic measurements including talo-calcaneal angle, talus-first metatarsal angle, and calcaneus-fifth metatarsal angle improved in all patients compared with preoperative values.^19^

A series of 16 patients with dorsal-lateral subluxation of the talonavicular joint and associated cavovarus deformity were reviewed by Wei et al^21^ The average age was 11 years (range 4 to 20 years), and all patients were treated with talonavicular fusion and medial release. Eight patients also required lateral column shortening. They reported good satisfaction and symptomatic improvement in this patient group. Ramseier et al^22^ reported on seven adult patients with recurrent (undercorrected) idiopathic clubfeet treated with triple arthrodesis. These patients were primarily in their fourth and fifth decades of life and reported good results.

Eidelman et al^27^ reviewed 11 adolescent patients who underwent midfoot osteotomy and Taylor spatial butt frame application to correct residual clubfoot at an average age of 14.7 years (range 11 to 18 years). Six patients had previously undergone posteromedial release, whereas 4 had complete soft-tissue release and 1 had undergone fasciotomies for compartment syndrome. Three patients had a diagnosis of arthrogryposis, whereas 1 had acquired clubfoot deformity as a sequela of compartment syndrome. All patients attained the desired deformity correction by the time of frame removal (average frame time was 15.1 weeks). Two patients required reoperation for relapse, and another was indicated for reoperation at a later date. The authors concluded that midfoot osteotomy and Taylor spatial butt frame can be used to correct stiff midfoot and forefoot deformities (forefoot adduction, supination, and cavus).^27^

El-Sayed^28^ evaluated the use of Ilizarov external fixation in the correction of 42 relapsed clubfeet in patients who underwent previous soft-tissue releases. Although the average age at the time of external fixation was 6 years, patients' age ranged from 3 to 13 years. Approximately half (20 feet) underwent ringed-external fixation alone, whereas 22 feet required additional soft-tissue release in conjunction with frame application. Outcomes were determined by the Beatson and Pearson numerical assessment scale, and good or excellent results were reported in 37 feet compared with poor results in five feet, at a mean follow-up of 4.6 years. Of note, younger patients and those who had undergone fewer previous surgeries tended to have better outcomes. Based on these results, El-Sayed^28^ considers the Ilizarov technique to be an acceptable treatment alternative for severe recurrent deformity.

Radler and Mindler^29^ provided a comprehensive review of different treatment strategies for severe recurrent clubfoot deformity in patients who underwent Ponseti casting and/or previous surgery. Treatment options were discussed according to different components of the residual deformity, including subtalar rotation, ankle equinus, cavus foot, heel varus, and forefoot adduction. For each component, the authors reviewed various bony and soft-tissue procedures but noted a current trend toward external fixation for the stiff clubfoot with severe deformity owing to the potential for gradual correction with minimal bony procedures.^29^ Although the authors included clinical photographss and radiographs, no results were reported.

In a recent review on the management of relapsed, residual, and neglected clubfeet, Eidelman et al^30^ discussed the challenges of selecting appropriate treatment strategies in this heterogeneous group. The authors divided relapses into the following 3 groups: early (from 6 to 30 months), older children (from 30 months to 8 years), and adolescents (9 years and older). They note that many patients in the adolescent group were treated with previous surgical procedures rather than the Ponseti method alone, although this number is steadily decreasing. They summarized their preferred method of treatment for rigid deformities that includes osteotomies and application of an external fixation frame for gradual correction. The authors prefer using a standard Taylor spatial frame for the correction of equinus in younger children and the butt frame, which includes a U-shaped plate around the foot, for the correction of midfoot and forefoot deformities.^30^

Sankar et al^31^ reported on the use of gait analysis to aid in the selection of corrective procedures for recurrent clubfoot deformity. The average age of patients in their study was 6.7 years (range 3.6 to 15.4 years). Thirty-five patients with 56 clubfeet were included, of which 41 had undergone previous posterior medial-lateral release. Using data from the computerized motion analysis, dynamic electromyography, and physical examination, the authors identified several common deformities in their cohort including the following: intoeing (80%), internal tibial torsion (71%), forefoot adductus (71%), and tibialis anterior over-activity (50%). The authors recommended specific procedures to correct the deformities identified by the gait analysis and compared these with pregait analysis surgical plans. In 19 of 30 patients who ultimately underwent surgery, these recommendations resulted in a total of 28 changed procedures, as compared to the surgical plans conceived before the gait analysis. The most frequent procedures in this series were split anterior tibialis tendon transfers (34), tibial derotational osteotomies (34), and midfoot osteotomies (20). The split anterior tibialis tendon transfer was recommended for hindfoot varus that occurred during inappropriate tibialis anterior activity on electromyography. Tibial derotational osteotomy was advised for tibial torsion, whereas midfoot osteotomy was recommended for fixed supination and internal rotation deformities not arising from tibial torsion.^31^

## Overcorrection

Other pathologies such as hindfoot valgus and forefoot abduction may be classified as overcorrection and are potential sequalae of soft-tissue release for clubfoot deformity.^18,23,24^ The literature on overcorrected clubfeet in adolescent patients is sparse. Knupp et al^23^ conducted a prospective study on 14 adult patients (average age of 37 years) with overcorrected clubfeet. All patients underwent a supramalleolar osteotomy to correct the deformity. The orientation of the tibial plafond was restored on radiographs in all patients. Statistically significant improvements were reported in the average visual analog pain score (from 4.1 to 2.2), American Orthopaedic Foot and Ankle Society hindfoot score (from 51.6 to 77.8), and ankle motion (from 25° to 29°). In addition, all patients had the ability to wear normal footwear. The authors concluded that a supramalleolar osteotomy can successfully treat ankle impingement, resulting from the overcorrection of clubfeet.^23^

Zide and Myerson^24^ reviewed their experience with treatment of the overcorrected clubfoot deformity. The authors considered dorsal bunion, dorsal navicular subluxation, and anterior ankle impingement to be varieties of overcorrection. For dorsal bunion deformities, the authors preferentially used anterior tibial tendon transfer and first tarsometatarsal joint arthrodesis to address the muscular imbalance and bony deformity, respectively. To treat dorsal navicular subluxation, they used talonavicular arthrodesis along with medial displacement calcaneal osteotomy for symptoms localized to the talonavicular joint. For navicular subluxation and symptoms involving the subtalar joint, the authors preferred triple arthrodesis. In treating anterior impingement, the authors have attempted cheilectomy, but this often failed because of concomitant ankle arthritis. They note that ankle arthrodesis is an option but not ideal because of the potential for development of adjacent joint arthritis in young patients. Although the idea of ankle arthroplasty was entertained, the chance of failure in young patients is high.^24^ No clinical outcomes were reported in this review article.

### Other Sequelae

Although several articles have focused on either relapse and recurrence or overcorrection, a few articles discussed other sequalae of previously operated clubfeet.^17,18,20^ Kuo and Smith^18^ reviewed 134 clubfeet in 85 children (aged 2 to 7.4 years) who underwent previous hindfoot release and identified common deformities including the following: forefoot adduction and supination, in-toeing gait, overcorrection, navicular rotatory dorsal subluxation, and dorsal bunion. From this cohort, 21 clubfeet underwent additional surgery. The most common indication for revision surgery was residual forefoot adduction and supination deformity, whereas the most common additional procedure was anterior tibial tendon transfer. The results of revision surgery were rated as excellent in 5 feet, good in 8 feet, and fair in 8 feet.^18^

Walling and Brodsky discussed their respective clinical experiences with adult patients with sequelae of treated congenital clubfoot, including clinical presentation, diagnosis, and treatment.^17,20^ Their reviews included the categories of undercorrection, overcorrection, arthritis, and degenerative conditions of the ankle and hindfoot. Both authors emphasize that adults with previous clubfoot treatment present with a variety of residual or recurrent deformities that must be considered in selecting appropriate treatment. Walling^20^ informs that flexible deformities may be treated with a combination of soft-tissue releases and osteotomies or limited fusions but more rigid feet often require more extensive fusions that may involve the ankle, subtalar, and talonavicular joints. Similarly, Brodsky found arthrodesis and osteotomy procedures to be most useful in these patients. With the exception of one case report, no clinical outcomes were reported.^17^

### Surgical Approaches to the Adolescent Clubfoot Deformity

Preoperative evaluation is critical to evaluate the presenting complaints and to create a rational surgical plan for management. Assessment of deformities, such as heel varus or valgus, residual cavus, or forefoot adductus, should be done in both the standing position and the seated position with the hindfoot held in neutral. The relationship between the hindfoot and the forefoot should be carefully examined to determine whether there is deformity between the hindfoot and forefoot and whether it is a flexible or fixed deformity. Weight-bearing CT was not available for evaluation of patients in our clinical series but would aid the surgeon in better understanding these 3-dimensional deformities, especially in the hindfoot.

It is important to determine the range of motion of the critical joints of the hindfoot which may require an examination under fluoroscopy to accurately determine where the primary motion is occurring. The ankle joint in many patients is very stiff, and compensatory range of motion through the talonavicular joint is often mistaken for ankle joint range of motion. The surgeon should be aware that a talonavicular arthrodesis in this setting would therefore eliminate a significant amount of dorsiflexion-plantarflexion range of motion. In addition, the lack of ankle joint dorsiflexion may not be improved with an Achilles tendon lengthening procedure, given the arthrofibrosis or bony impingement at the tibiotalar joint. Careful examination of muscle strength is critical in looking for imbalances such as overpull of the anterior tibialis muscle as a deforming force for the dorsal bunion deformity. Previous over lengthening of the gastroc-soleus complex may require tendon transfers of the peroneals or the toe flexors to the calcaneus to restore the plantar flexor power. The location of previous incisions about the foot and ankle should be taken into account when planning future reconstructive procedures to reduce the potential for wound healing complications.

Only through careful history and physical examination can the surgeon determine the primary reasons for pain in the adolescent clubfoot because radiographically many of the feet have significant abnormalities but often function at a high level despite their radiographic deformity.

The primary goal of the treatment is to obtain a plantigrade foot through osteotomies, soft-tissue balancing, and gradual distraction with external fixation or arthrodesis and not necessarily correct all abnormal radiographic angles and joint alignments. The second goal is to eliminate sources of pain which are often related to asymmetrical loading of a nonplantigrade foot or bony prominences, or painful arthritic joints. A third goal is to maintain as much functional range of motion as possible, given the significant stiffness that is often observed at the time of presentation. Therefore, periarticular osteotomies and soft-tissue balancing procedures are favored over arthrodesis with the goal to maintain range of motion at the primary joints of the foot and ankle. Given the varied presentations of these patients, a wide spectrum of different surgical procedures are required to manage the presenting problems as evidenced by the listing of procedures in Appendices 1–5, http://links.lww.com/JG9/A74.

## Our Clinical Series Review

### Overcorrection

The problem of overcorrection of the clubfoot deformity represented the largest group in our study (25 patients, 37 feet). The primary presenting problems were related to severe heel valgus, lateral translation of the calcaneus relative to the talus, and fixed forefoot abduction. To address this deformity, 66 procedures were performed in this group (1.78 procedures per foot). If the deformity was flexible, a more traditional pes planovalgus deformity correction procedure was performed with either a lateral column lengthening or medial displacement calcaneal osteotomy or both, coupled with a soft-tissue advancement with FDL tendon transfer as necessary. The more severe deformity with limited flexibility often required a more complex procedure with a limited arthrodesis such as a lateral column lengthening calcaneal-cuboid joint fusion with medial displacement calcaneal osteotomy and medial soft-tissue reefing of the spring ligament complex, and a medial column midtarsal arthrodesis for correction of residual deformity. A complete listing of the procedures required to manage this group of patients are listed in (Appendix 1, http://links.lww.com/JG9/A74).

### Undercorrection

Undercorrection of the clubfoot deformity affected 25 patients (28 feet) in our clinical series. The undercorrected clubfeet often had residual heel varus and cavus deformity with forefoot adduction or fixed forefoot varus and more stiffness of the hindfoot than in the overcorrected patients. The primary procedures to correct these deformities were valgus producing closing or lateral sliding calcaneal osteotomies with midfoot osteotomies to derotate the forefoot out of varus and also correct adduction or cavus (Appendix 2, http://links.lww.com/JG9/A74). For the mild to moderate deformities, a single-staged correction was performed. However, there were several severe fixed deformities which required a computer-assisted multiplanar external fixation device for gradual correction of the forefoot varus deformity. Soft-tissue balancing was often required with anterior tibialis tendon transfer to the second or third cuneiform.

When the residual cavovarus deformity was primarily localized in the midfoot, a midfoot biplanar transverse osteotomy, often with an allograft medial wedge, was done along with peroneus longus to brevis transfer and anterior tibialis tendon transfer to the third cuneiform. Additional metatarsal osteotomies were also added when necessary. Overall, 76 procedures were required in this group of patients for an average of 2.81 procedures per foot.

### Dorsal Bunion

The dorsal bunion deformity was the primary presenting problem in nine patients (12 feet) in our clinical series (Appendix 3, http://links.lww.com/JG9/A74). These deformities were exhibited by elevation of the medial column with overpull of the anterior tibialis muscle combined with a weak peroneus longus muscle. In addition, there was also compensatory overpull of the flexor hallucis longus (FHL) and flexor hallucis brevis (FHB) with flexion at the first metatarsophalangeal (MTP) joint. Hindfoot valgus and dorsal rubbing of the first metatarsal head with footwear was often associated. Radiographically, most of the deformity was primarily located at the naviculocuneiform joint or talonavicular joint area. Therefore, a naviculocuneiform joint arthrodesis procedure with interposition of allograft bone wedges into the naviculocuneiform joints was the primary treatment of choice for the more severe deformities. Correction of the medial column bony deformity, more distal to the naviculocuneiform joint such as with cuneiform or first metatarsal osteotomies, is not as powerful at correcting this deformity. The double bone-block fusion of the naviculocuneiform and the 1 to 2 intercuneiform joint has been described previously and addresses the dorsal bunion closer to the apex of the deformity.^24^ This procedure was often combined with anterior tibial tendon transfer to the second or third cuneiform with intermuscular lengthening of the flexor hallicus brevis with MTP joint capsular release. FHL transfer to the first metatarsal neck was also often performed.^24^ Correction of the dorsal bunion required 36 procedures in this group, which represented three procedures per foot at the time of reconstruction.

### Anterior Ankle Impingement

Anterior ankle impingement with limited ankle joint dorsiflexion range of motion occurred in 10 patients (13 feet) in our clinical series. These patients often had a combination of ankle joint equinus with reduction of the normal lateral distal tibial articular angle and loss of the normal curvature of the talar dome. Many ankles had significant arthrofibrosis and early osteoarthritis. Management options included dorsiflexion osteotomy of the distal tibia or excision of bone causing anterior ankle impingement either from the dorsal lip of the navicular or the anterior aspect of the distal tibia or both (Appendix 4, http://links.lww.com/JG9/A74). Occasionally, Achilles tendon lengthening was helpful to improve dorsiflexion range of motion. However, in most cases limited dorsiflexion was not related to an Achilles contracture, but rather anterior impingement. Anterior ankle joint arthroscopic soft-tissue débridement was used later in the series in combination with dorsiflexion closing-wedge osteotomy of the distal tibia. Eighteen procedures were required in this group for a total of 1.38 procedures per foot. Anterior tibial epiphyseal plate modulation was used in younger patients.

### Lateral Impingement

The final category of presenting problems in our clinical series related primarily to lateral impingement either from an exostosis on the lateral wall of the calcaneus, soft-tissue sinus tarsi syndrome, or calcaneal fibular abutment (Appendix 5, http://links.lww.com/JG9/A74). A preoperative CT scan was helpful in planning the surgical procedure. In many cases the lateral wall of the calcaneus was hypertrophic, and a shelf of calcaneal bone extended under the tip of the fibula with or without associated lateral translation of the calcaneus. In these patients, who otherwise had a plantigrade foot, a lateral wall or distal fibular exostectomy was performed, accounting for five procedures in four limbs (1.25 procedures per limb).

## Discussion

In contrast with the long-term results of the Ponseti method of treatment, patients with a history of clubfoot deformity and previous extensive soft-tissue release surgeries on young feet leads to poor long-term outcomes with many patients having multiple procedures through childhood and adolescence. Our case series demonstrates the variety of residual deformities present in this population, for which there are no clear-cut treatment protocols. However, patterns of deformity exist and may help guide treatment. Over two-thirds of the patients in this series presented with history and examination findings consistent with undercorrection or overcorrection of the deformity, whereas bony impingements and dorsal bunions were present in lesser numbers. Careful physical examination and radiographic evaluation are critical for developing a surgical plan.

The strength of this report is in the comprehensive review of the variable presentations of the adolescent patient who is having residual problems with their clubfoot, and it fills a void in the literature regarding this topic. The information presented provides surgeons with a framework for evaluation and classification, describes the goals and principles of treatment, and provides a list of different procedures that were used in this group of patients to correct their deformity during the index surgery at our institution.

Limitations in our case series include the retrospective design, our heterogeneous study population, and the difficulty in obtaining meaningful outcomes data on enough patients to report the results of our treatment. Many patients travel long distances to receive care at our regional referral center, and given the 13-year time span of the treatments, obtaining a follow-up is difficult. Despite these limitations, a review of the clinical experience of five surgeons within two closely related institutions of a regional referral center serves to inform our classification system and develop recommendations for treatment.

## Conclusion

Identifying patterns of pathology present in adolescent and young adult patients can aid in the evaluation and treatment of complex residual clubfoot deformities. This case series and literature review may help develop a more standardized approach to the treatment of these difficult problems with the goal of maintaining motion, improving function, and decreasing pain. Separation of patients into diagnostic categories with a listing of the surgical procedures needed to correct these deformities may help surgeons plan these complex procedures and guide future research focused on this group of patients. The Ponseti Method has proven long-term results into adulthood and it is hoped that because this method is adopted in more parts of the world, there will be fewer long-term sequelae requiring surgical treatment.^12-14^
